# A Fiducial-Marker-Based Localization Method for Automotive Chassis Bolt Assembly

**DOI:** 10.3390/s26061818

**Published:** 2026-03-13

**Authors:** Xiangqian Peng, Yingjie Xiao, Zhewu Chen, Kaijie Chen, Hong Huang

**Affiliations:** 1 School of Mechanical Engineering, Hunan University of Science and Technology, Xiangtan 411201, China; 17674543407@163.com (Y.X.); chenzhewu@126.com (Z.C.); 18163951241@163.com (K.C.); 2 School of Intelligent Science and Engineering, Hunan Engineering University, Xiangtan 411104, China; huanghong712@126.com

**Keywords:** automotive chassis bolt assembly, visual localization method, Retinex model, ellipse fitting, Leitz criterion

## Abstract

To address the difficulty of accurately localizing automotive chassis bolts during the assembly process—caused by non-uniform illumination, limited camera installation space, and occlusions from the vehicle body structure—a fiducial-marker-based localization method is proposed. In this method, a concentric ring-shaped fiducial marker is affixed to the bottom of the assembly wrench, and its region of interest (ROI) is extracted using an HSV color space segmentation algorithm. To overcome interference from uneven lighting and insufficient brightness in industrial environments, an improved Retinex-based image enhancement algorithm is introduced, which significantly improves the robustness and accuracy of ROI extraction. The extracted ROI image is subjected to ellipse fitting, and the fitting process is optimized by incorporating the Leitz criterion. Experimental results show that the optimized ellipse fitting algorithm achieves higher accuracy and significantly enhances the reliability of fitting. Since perspective projection of spatial circles leads to displacement of the circle center, the actual projected center of the fiducial marker in the image is calculated by estimating the normal vector of the circular plane using vanishing lines and the ellipse parameter matrix. This enables spatial localization of the bolt end. The proposed method is validated by comparing the localization results with the theoretical coordinates of the bolt holes. Experimental results demonstrate that the method offers high localization accuracy and strong robustness, meeting the practical precision requirements for automatic bolt assembly in industrial applications.

## 1. Introduction

In the automotive manufacturing process, the chassis serves as a key load-bearing structure with a complex design, requiring extremely high connection strength and assembly precision. A large number of bolts with varying specifications and complex positional distributions are used for fixation. Traditional assembly methods rely on manual confirmation of the bolt type, installation position, and corresponding torque-angle parameters based on process documentation, with assembly operations completed using a manual torque wrench. This process is not only labor-intensive and inefficient but also highly susceptible to human error, making it difficult to ensure assembly quality. With the development of industrial automation, the integration of bolt localization systems based on 3D stereoscopic vision technology with assembly equipment provides a new solution to this issue.

In automotive chassis bolt localization systems, accurate bolt localization presents significant challenges due to factors such as non-uniform illumination, limited camera installation space, and occlusions from vehicle body structures. Furthermore, each chassis image contains multiple bolts, with each bolt occupying a small area, which complicates precise localization. To address this issue, this paper proposes a fiducial-marker-based localization method for automotive chassis bolt assembly. A concentric ring-shaped fiducial marker is affixed to the bottom of the wrench, and precise localization of the automotive chassis bolt is achieved through the accurate localization of the marker’s center. While there has been no research on using fiducial markers for chassis bolt localization under a vehicle, extensive studies have been conducted internationally on fiducial markers in other application scenarios. For instance, Michail Kalaitzakis et al. [1] comprehensively compared the performance of common square markers (ARTag, AprilTag, ArUco, and STag) under various conditions. Their study shows that while square markers such as AprilTag exhibit strong robustness in occlusion scenarios, their pose estimation accuracy may degrade under extreme observation angles or non-uniform lighting in constrained industrial spaces. Lilian Calvet et al. [2] proposed a novel fiducial marker system called CCTag (Concentric Circle Tag), designed to address the failure of existing markers, especially square markers like AprilTag and simple circular markers, under adverse conditions such as motion blur, large defocus, low resolution, and non-uniform lighting. The concentric circle structure in the CCTag, which becomes concentric ellipses under perspective projection, possesses excellent geometric invariance properties. Tomáš Pivoňka et al. [3] developed a robust and accurate robot docking system using monocular vision and AprilTags, along with automatic calibration based on SfM, highlighting the algorithm’s ability to replace cumbersome manual environment measurements and significantly reduce deployment and maintenance barriers. Filippo Bergamasco et al. [4] proposed a new fiducial marker system called Rune-tag, based on a dot-array concentric circle design, which offers high occlusion resistance, accuracy, and projective invariance. The concentric ring fiducial marker used in this paper inherits the geometric invariance advantages of CCTag and Rune-tag, but applying CCTag and Rune-tag directly to chassis bolt localization still faces specific limitations. For example, while they excel at handling noise, they do not address the issue of feature extraction failure due to non-uniform lighting on the chassis. Additionally, most circular-marker-based localization methods focus on detection robustness but often overlook geometric eccentricity errors, which are critical in high-precision localization tasks. To overcome these challenges, this paper introduces improvements to existing techniques. Huang et al. [5,6,7] proposed a low-light image enhancement algorithm based on the Retinex theory to address the issue of imprecise bolt localization due to uneven lighting and low-light conditions in automotive chassis images. This method separates the illumination and reflection components of low-light images and enhances image details under low-light conditions through adaptive gain adjustment and color correction. In this paper, Huang et al.’s method, combining an improved Retinex algorithm with HSV segmentation, stably extracts the fiducial marker even under harsh, uneven industrial lighting conditions. Yang et al. [8] proposed an adaptive sub-pixel edge detection algorithm for locating nut hole centers, which analyzes pixel intensity distribution features of hole edges under Gaussian-weighted adaptive thresholds, and achieves precise automatic center determination using least squares circle fitting in noisy environments. Additionally, Wang et al. [9] proposed an ellipse parameterized geometric fitting method to address the low fitting accuracy of bolts under partial occlusion. This method analyzes the coupling relationships between ellipse geometric parameters and the calculation of geometric distance errors, using an improved least squares optimization algorithm to achieve high-precision fitting of industrial component elliptical profiles under high noise and partial occlusion conditions. This paper introduces the Leitz criterion to optimize ellipse fitting, achieving better fitting quality than the standard least squares method used in previous works. Since perspective projection of spatial circles causes displacement of the circle center, thus reducing bolt localization accuracy, Cui et al. [10,11] proposed a high-precision localization algorithm for the center of circular targets in visual measurement. By analyzing the geometric causes of center displacement during ellipse fitting and introducing corresponding correction methods, iterative optimization and sub-pixel edge extraction were used to achieve high-precision localization of circular target centers under complex angles and lighting conditions. This paper calculates the normal vector of the spatial circle plane and, using vanishing lines and the ellipse parameter matrix, computes the actual projected center of the concentric ring fiducial marker in the image, compensating for the marker’s shortcomings in lighting adaptability and projection deviation correction.

This paper improves and integrates existing technologies, successfully overcoming the challenges of automotive chassis bolt localization caused by non-uniform illumination, limited camera installation space, and occlusions from the vehicle body structure. It achieves high-precision localization of automotive chassis bolts, meeting the practical precision requirements for bolt assembly in industrial applications.

## 2. Feature Extraction of Circular Targets

In the automatic assembly process of automotive chassis bolts, spatial localization of the bolt end is primarily achieved by acquiring the coordinates of the concentric circular fiducial marker affixed to the bottom of the assembly wrench. This process involves capturing images of the concentric circular fiducial from the bottom of the wrench using two cameras positioned on the left and right sides. The region of interest (ROI) corresponding to the fiducial marker is then extracted using an HSV color space segmentation algorithm. Subsequently, a series of image processing steps—including binarization, edge detection, contour linking, and ellipse fitting—are performed on the extracted ROI image to extract the contour features of the concentric circular fiducial marker.

### 2.1. HSV-Based Color Segmentation Algorithm for Target Extraction

In the current industrialized automotive production process, the chassis structure is typically large in volume. To ensure that the designed camera system can capture the entire chassis area, a wide field of view must be configured. As a result, the circular fiducial marker affixed to the bottom surface of the assembly wrench occupies only a small portion of the acquired image. Therefore, a preliminary localization of the fiducial marker is required to extract its ROI, which facilitates subsequent processing [12].

This study employs an HSV color segmentation algorithm to identify the purple region of the target, thus achieving the initial localization of the target. The HSV color space consists of three components: Hue, Saturation, and Value. Compared to the traditional RGB color space, the HSV color space aligns more closely with human visual perception, providing a more intuitive representation of the color tone, brightness, and saturation of an image. As a result, it offers distinct advantages in color-based segmentation tasks [13].

Since images displayed on electronic devices are typically encoded in the RGB format, and the RGB channels are not well-suited for representing specific color characteristics, it is often necessary to convert RGB components into HSV components for more effective color processing. The conversion relationships [14] are defined as shown in Equation (1).

(1) $$\left\{\begin{array}{l} R=R/255,G=G/255,H=H/255\\ V=\max\left(R,G,B\right)\\ S=\left\{\begin{array}{l} \frac{V-\min(R,G,B)}{V},(V\neq 0)\\ 0,(V=0) \end{array}\right.\\ H=\left\{\begin{array}{l} \frac{60{}^{\circ}(G-B)}{V-\min(R,G,B)},(V=R)\\ 120{}^{\circ}+\frac{60{}^{\circ}(B-R)}{V-\min(R,G,B)},(V=G)\\ 240{}^{\circ}+\frac{60{}^{\circ}(R-G)}{V-\min(R,G,B)},(V=B)\\ 0,(R=G=B) \end{array}\right. \end{array}\right.$$

By setting appropriate value ranges for the H, S, and V components, specific colors can be effectively identified, enabling the initial localization of the target. Based on the actual lighting conditions in the industrial environment, a well-illuminated target image with preserved details was captured. The image was processed using MATLAB (R2022b) to adjust the ranges for H, S, and V. When the range of H was [120°,160°], the range of [0.31,1] was S, and the range of V was [0.28,1], only the purple region corresponding to the concentric ring target on the bottom plane of the assembly wrench was detected. Therefore, the value ranges for H, S, and V were determined for subsequent processing.

### 2.2. Image Enhancement Processing:

**Figure 1:**
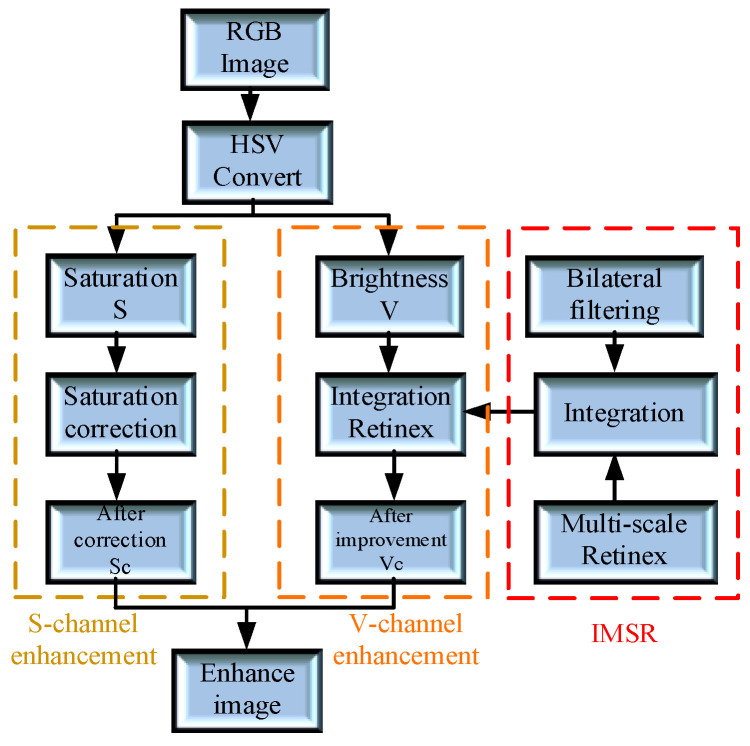
Image Enhancement Procedure Diagram.

In industrial environments, non-uniform illumination frequently occurs on automotive chassis surfaces, particularly in edge regions where lighting intensity is severely insufficient. Additionally, due to the large size of the chassis structure, image noise interference is significant, making it difficult to preserve the detailed features of the fiducial marker in poorly lit areas. This adversely affects subsequent visual observation and feature extraction. Under such conditions, the previously defined H, S, and V value ranges are insufficient for completely extracting the purple region of the circular fiducial marker. To address this issue, a Retinex-based image enhancement algorithm incorporating bilateral filtering is applied to enhance the fiducial marker image, thereby improving the visibility and contrast of the target region [15]. The overall image enhancement process is illustrated in Figure 1.

#### 2.2.1. Improved Retinex Algorithm

The Retinex model is based on the human visual system’s color constancy hypothesis [16], which is used to decompose an observed image into illumination and reflection components, thereby enhancing image details and contrast under non-uniform lighting conditions. The single-scale Retinex algorithm is simple and effective, but it performs poorly in enhancing image details. To better capture and enhance structures and details at various scales within the image, a multi-scale Retinex algorithm is proposed, with the following expression:

(2) $$r(x,y)=\sum_{k=1}^{N}w_{k}[\ln I(x,y)-\ln I((x,y)*G(x,y))]$$

Here, *N* represents the total number of scales, and $w_{k}$ denotes the weight assigned to each scale. When *N* = 3, the weights are typically set as *w*_1_ = *w*_1_ = *w*_3_ = 1/3; when *N* = 1, the multi-scale Retinex algorithm reduces to the single-scale Retinex algorithm.

In the Retinex algorithm, when estimating the illumination component using a Gaussian kernel, the scale parameter σ is highly sensitive to the enhancement effect: a large σ leads to insufficient contrast, while a small σ tends to produce halos and local distortions, which is particularly evident in industrial scenes with strong reflections, occlusions, and noise interference [17]. To improve edge preservation and suppress halo effects, this paper uses an improved bilateral filter to replace the Gaussian kernel as the central surrounding function for estimating the illumination component [18], with the weight function expressed as follows:

(3) $$G(x,y)=\exp[-\frac{{\left(x-x_{c}\right)}^{2}+{\left(y-y_{c}\right)}^{2}}{2\sigma_{s}^{2}}]\times \exp[-\frac{{\left(f(x,y)-f(x_{c},y_{c})\right)}^{2}}{2\sigma_{r}^{2}}]$$

In the equation, $\left(x_{c},y_{c}\right)$ denotes the position of the image center, and $f(x_{c},y_{c})$ represents the grayscale intensity of the pixel at the center. $\sigma_{s}$ is the standard deviation of the spatial-domain Gaussian function, and $\sigma_{s}$ is the standard deviation of the range-domain Gaussian function.

Substituting Equation (3) into Equation (2) yields the multi-scale Retinex algorithm based on bilateral filtering theory. The logarithmic transformation is then applied to r(x,y), resulting in the reflection image R(x,y).

#### 2.2.2. A Saturation Enhancement Algorithm Based on Contrast Stretching

When the hue component (H) remains unchanged, the overall visual effect of the image is primarily determined by the brightness and saturation. To enhance brightness while preserving edge structure, this paper introduces bilateral filtering to the brightness channel (V), resulting in clearer edge transitions. However, under conditions of insufficient or uneven illumination, images often exhibit low color vibrancy and reduced saturation levels. Enhancing brightness and details in such low-light scenarios may introduce color distortions if saturation is not properly adjusted. To mitigate this, a linear contrast stretching strategy is employed to optimize the saturation component, ensuring that its values are distributed effectively within the full dynamic range of [0, 255]. The computation of contrast stretching is given by Equation (4).

(4) $$S_{out}=\frac{S_{in}-L}{H-L}$$

In the equation, $S_{in}$ denotes the input saturation component, while L and H represent the lower and upper bounds of the saturation stretching range, respectively.

### 2.3. Procedures for Target Marker Feature Extraction

Under actual industrial lighting conditions, an initial fiducial marker image with uniform illumination and clear detail is first captured. MATLAB is used to perform color space processing on the image, and the HSV value ranges across the three channels are extracted as reference standards for subsequent feature extraction. The fiducial image to be processed is then subjected to color space conversion and channel separation; the RGB image is decomposed into three channels and transformed into the HSV space. To mitigate the effects of non-uniform illumination, an improved multi-scale Retinex algorithm is applied to enhance the brightness (V) channel, thereby improving both image detail and overall luminance. In addition, linear contrast stretching is performed on the saturation (S) channel to enhance the color expressiveness of the image and improve robustness under complex lighting conditions. Pixel-level analysis is conducted on the enhanced image: if a pixel’s HSV values fall within the predefined reference range, it is marked as white, resulting in a binarized image of the fiducial’s purple region. Finally, a minimum bounding rectangle is used to enclose all white regions in the binarized image, and the area covered by this rectangle is defined as the final region of interest (ROI) to be extracted.

**Figure 2:**
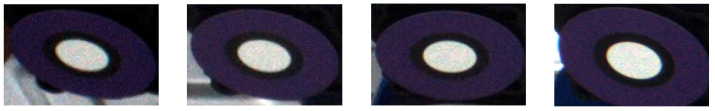
Original Image.

**Figure 3:**
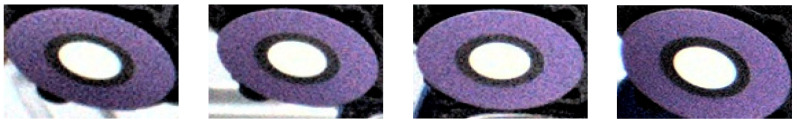
Image After Enhancement.

**Figure 4:**
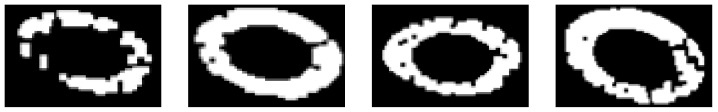
Binarization of the Original Image.

**Figure 5:**
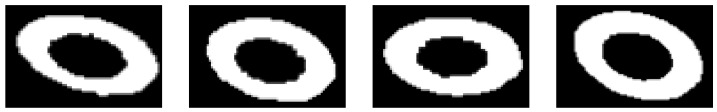
Binarized Image After Enhancement.

**Figure 6:**
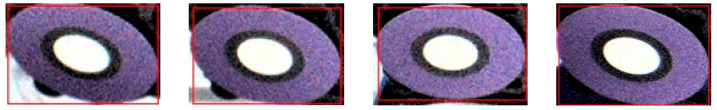
Region of Interest (ROI) of the Target Marker.

To verify the effectiveness of the proposed method, four fiducial marker images captured under low and uneven lighting conditions beneath the automotive chassis were selected, as shown in Figure 2. Image enhancement was applied to each of the original images, and the enhanced results are presented in Figure 3. Subsequently, the purple regions were extracted from both the original and enhanced images. The binarized image of the purple region from the original image is shown in Figure 4, while that from the enhanced image is shown in Figure 5. The final extracted regions of interest (ROI) are illustrated in Figure 6.

As shown in the figures below, the image enhancement process significantly improves overall image quality. Figure 2 shows the original image, in which the target object is visually merged with the background due to poor lighting conditions. This results in low contrast and blurred edges, making it difficult to distinguish the target from the background. Consequently, the corresponding binarized result in Figure 4 contains substantial noise artifacts and incomplete representation of the target object, hindering effective extraction of the target region. In contrast, Figure 3 displays the enhanced image, where the target object appears clearly defined, with strong contrast against the background and well-preserved edge details. These improvements facilitate more accurate feature extraction, as evidenced by the binarized result shown in Figure 5, which exhibits clear contours, complete structure, and distinct separation between the foreground target and the background, allowing the region of interest (ROI) of the fiducial marker to be effectively extracted. These results confirm that the proposed improved multi-scale Retinex image enhancement algorithm effectively addresses the difficulties in feature extraction caused by uneven illumination, especially in the poorly lit edge areas of the automotive chassis. The method enables stable and complete extraction of the fiducial marker’s purple region.

### 2.4. Canny Edge Detection Applied to the Region of Interest

**Figure 7:**
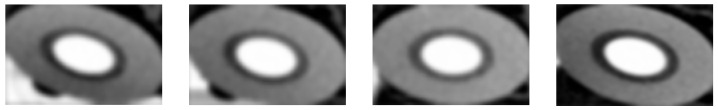
Gaussian-Filtered Image of the Circular Ring.

The region of interest (ROI) of the fiducial marker, extracted using the HSV color space segmentation algorithm, undergoes Canny edge detection. Compared to direct thresholding or simple gradient operators, Canny edge detection is more suitable for subsequent contour extraction and ellipse fitting, as it performs better in noise suppression, edge localization, and edge continuity. The algorithm process includes: Gaussian smoothing—gradient calculation—non-maximum suppression—double-threshold hysteresis. The image after Gaussian filtering of the ROI is shown in Figure 7.

In the Canny algorithm process, the selection of dual thresholds directly impacts the edge continuity and noise resistance of Canny edge detection. Traditional approaches rely on empirically set high and low thresholds, which are difficult to adapt to industrial scenes with non-uniform illumination. This paper introduces the Otsu algorithm to enable adaptive selection of high and low thresholds during edge linking, thereby effectively improving the stability and continuity of edge detection [19].

For an image of size *M* × *M*, with a grayscale range of [0,L−1], the pixels are divided into two classes, *C*0 and *C*1, using a threshold *t*. The probabilities and means of these classes are defined as shown in Equation (5).

(5) $$\left\{\begin{array}{l} P_{0}(t)=\sum_{i=1}^{t}P_{i}\\ P_{1}(t)=\sum_{i=t+1}^{L-1}P_{i}=1-P_{0}(t)\\ u_{0}(t)=\sum_{i=0}^{t}\left(i\frac{P_{i}}{P_{0}(t)}\right)\\ u_{1}(t)=\sum_{i=t+1}^{L-1}\left(i\frac{P_{i}}{P_{0}(t)}\right) \end{array}\right.$$

Based on the above results, the expression for the between-class variance of the image is given by Equation (6):

(6) $$\sigma (t)=P_{0}(t)u_{0}^{2}(t)+P_{1}(t)u_{1}^{2}(t)$$

Through iterative computation, the segmentation threshold corresponding to the maximum between-class variance is identified as the optimal threshold. At this point, the low threshold is denoted as $T_{l}=T_{h}/2$, and the high threshold $T_{h}$ is defined by Equation (7).

(7) $$T_{h}=\arg\max\{\sigma (t)\}=\arg\max\{P_{0}(t)u_{0}^{2}(t)+P_{1}(t)u_{1}^{2}(t)\}$$

**Figure 8:**
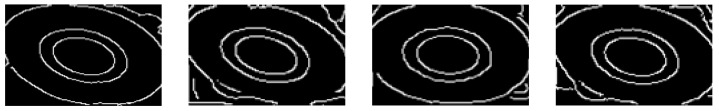
Image Processed by Canny Edge Detection.

The Canny algorithm described above is applied to perform edge detection on the region of interest shown in Figure 6. The detected adjacent edge points are subsequently connected to form contours, as illustrated in Figure 8.

As shown in Figure 8, the Canny edge detection algorithm employed in this study effectively extracts the boundaries of the circular fiducial marker. The detected edge contours are continuous and clear, exhibiting good structural integrity. Although minor discontinuities are observed near the edges in some images, the overall structural features of the target object are well preserved. These results indicate that the Canny edge detection algorithm used in this work provides high edge detection accuracy.

### 2.5. Ellipse Fitting Performed Using the Least Squares Method

In practice, the marker affixed to the bottom surface of the assembly wrench consists of a concentric circular ring structure. However, due to the angular deviation between the imaging plane of the camera and the bottom surface of the wrench, the projected circular marker appears elliptical in the captured image. Therefore, ellipse fitting is performed on the region of interest (ROI) of the circular marker using the least squares method.

The least squares method is an optimal estimation technique derived from the maximum likelihood estimation principle, under the assumption that the random errors of the sample measurements follow a normal distribution [20]. The fundamental constraint of this method is to minimize the sum of algebraic distances between all sample points and the fitted curve. Let $M(x_{0},y_{0})$ be a point in the plane and f(x,y)=0 the equation of the curve, then $f(x_{0},y_{0})$ represents the algebraic distance from point M to the curve. By applying the least squares method to the set of discrete sample points, an objective function for ellipse fitting is constructed, as shown in Equation (8). The coefficients of the ellipse equation are determined by minimizing this objective function.

(8) $$f(A,B,C,D,E)={\sum_{i=1}^{n}\left(Ax_{i}^{2}+Bx_{i}y_{i}+Cy_{i}^{2}+Dx_{i}+Ey_{i}+F\right)}^{2}$$

According to the extremum principle, in order to minimize f(A,B,C,D,E,F), it is only necessary to satisfy the following condition:

(9) $$\frac{\partial f}{\partial A}=\frac{\partial f}{\partial B}=\frac{\partial f}{\partial C}=\frac{\partial f}{\partial D}=\frac{\partial f}{\partial E}=\frac{\partial f}{\partial F}=0$$

A linear system of equations is thereby obtained, from which the coefficients *A*, *B*, *C*, *D*, *E* and *F* of the ellipse equation can be solved using the full pivoting Gaussian elimination method, in conjunction with the imposed constraint conditions.

Based on the geometric properties of ellipses, five geometric parameters of the ellipse can be calculated, including the center position $\left(x_{c},y_{c}\right)$, the lengths of the semi-major and semi-minor axes (a,b), and the orientation angle of the major axis θ. In a two-dimensional plane, any ellipse can be fully characterized by these parameters.

### 2.6. Ellipse Fitting Algorithm Optimized by the Leitz Criterion

According to Gaussian error theory, when measurement values follow a normal distribution, the probability of the residuals falling within three times the standard deviation, i.e., within the [−3σ,3σ] interval, is greater than 99.7%, while the probability of falling outside this interval is less than 0.3%. Therefore, measurement values whose residuals fall outside this region can be considered outliers [21]. This is the Leitz criterion for outlier detection, which is also commonly referred to as the 3σ method.

When performing least squares fitting on discrete sample points, the presence of outliers in the sample points can significantly affect the curve fitting results. Such points should be excluded from the curve to be fitted. In this paper, the Leitz criterion is used to remove outliers from the sample points.

Using the objective function defined in Equation (8), the fitted ellipse in the pixel coordinate system yields its center $o^{\prime }(x_{c},y_{c})$, major axis *a*, minor axis *b*, and orientation angle θ. Let N be the number of edge points used in the fitting. The coordinates of each edge point can be obtained using OpenCV functions. Denoting the coordinates of an edge point as $p(x_{i},y_{i})$, the algebraic pixel distance between this point and the ellipse center is then calculated accordingly:

(10) $$l(i)=\sqrt{{\left(x_{i}-x_{c}\right)}^{2}+{\left(y_{i}-y_{c}\right)}^{2}};\;i=1,2,\ldots ,N$$

Perform data analysis on the sample point set l(i) based on the Leitz criterion, removing the error points present in l(i). According to the Leitz criterion, the arithmetic mean of the sample point set is first calculated:

(11) $$\bar{L}=\frac{1}{N}\sum_{i=1}^{N}l(i);\;i=1,2,\ldots ,N$$

Subsequently, the standard deviation σ is calculated:

(12) $$\sigma =\sqrt{\sum_{i=1}^{N}\frac{{\left(l(i)-\bar{L}\right)}^{2}}{N-1}};\;i=1,2,\ldots ,N$$

The residuals of the data set are then calculated as follows:

(13) $$l_{b}(i)=l(i)-\bar{L};\;i=1,2,\ldots ,N$$

According to the Leitz criterion, if $l_{b}\leq -3\sigma$ or $l_{b}\geq 3\sigma$, then point i is considered an outlier. Using the correlation function, this point is excluded from the curve to be fitted. The least squares method is then applied to perform ellipse fitting on the new curve to be fitted. This process is repeated until the residuals of l(i) fall within $l_{b}(i)$, indicating that there are no outliers in the data set. The final fitted ellipse is the optimal ellipse.

**Figure 9:**
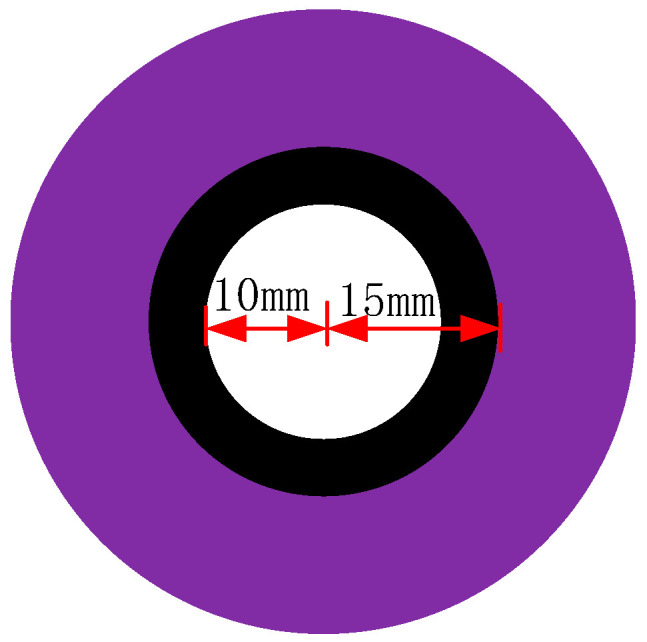
Dimensions of the Target Marker.

**Figure 10:**
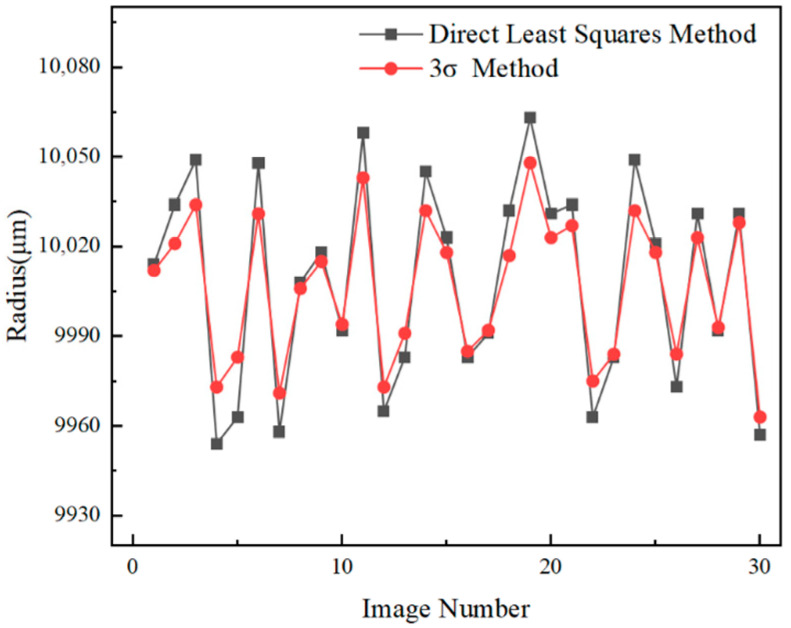
Estimated Radius of the White Circle.

**Figure 11:**
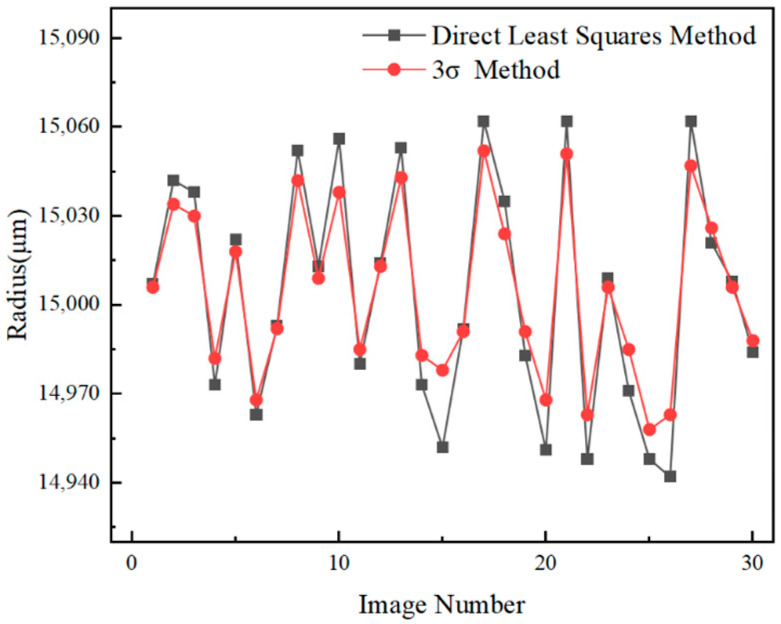
Estimated Radius of the Black Ring.

An error analysis was conducted on the estimated contour radius of the circular marker using computational algorithms. The designed circular marker consists of a white circle with a radius of 10 mm and a black ring with a radius of 15 mm. The detailed dimensions of the marker are shown in Figure 9. Under actual industrial conditions, 30 images of the circular marker were captured. Two different ellipse fitting algorithms were applied to each image to fit the circular marker. Using a binocular vision imaging model, the coordinates of all ellipse edge points and the ellipse center were computed in the camera coordinate system. For the white circle, the Euclidean distances from all fitted edge points to the ellipse center were calculated, and their average was taken as the estimated radius. The estimated radius of the white circle is shown in Figure 10. The same method was applied to estimate the radius of the black ring, with the result shown in Figure 11. The standard deviation, mean error, maximum error, and minimum error of the estimated radii were computed for both the white circle and black ring. The final error analysis results are summarized in Table 1.

As shown in Table 1, the ellipse fitting algorithm optimized using method 3σ results in significant reductions in the standard deviation, mean error, and peak error of the two estimated radii. This demonstrates that the algorithm effectively reduces ellipse fitting errors and substantially improves fitting accuracy.

**Figure 12:**
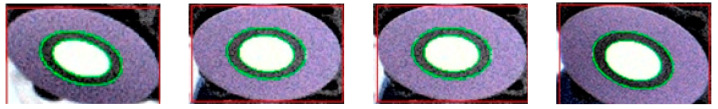
Ellipse Fitting Results.

The ellipse fitting algorithm optimized by the Leitz criterion using the least squares method is applied to the region of interest in Figure 6. The fitting result is shown in Figure 12. It can be observed that the algorithm accurately fits the contour of the circular marker and yields five geometric parameters: the ellipse center position $\left(x_{c},y_{c}\right)$, the lengths of the semi-major and semi-minor axes (a,b), and the orientation angle of the major axis θ. These parameters lay the foundation for the subsequent spatial positioning of the bolt.

## 3. Precise Localization of the Spatial Circle Center

During image acquisition of the circular fiducial marker, the principal optical axis of the camera is not necessarily perpendicular to the plane of the marker. As a result, the spatial circle appears as an ellipse in the image due to perspective projection. It is important to note that the center of the resulting ellipse does not correspond to the true projected position of the spatial circle center in the image; a deviation exists between the two [22]. Based on the method proposed in [23], an improved approach is adopted in this study. The ambiguity in the calculation of the normal vector of the circular plane caused by dual solutions is resolved using binocular vision. Furthermore, the actual projected center of the spatial circle is determined by incorporating vanishing lines and the ellipse parameter matrix.

### 3.1. Analysis of Spatial Circle Center Deviation

**Figure 13:**
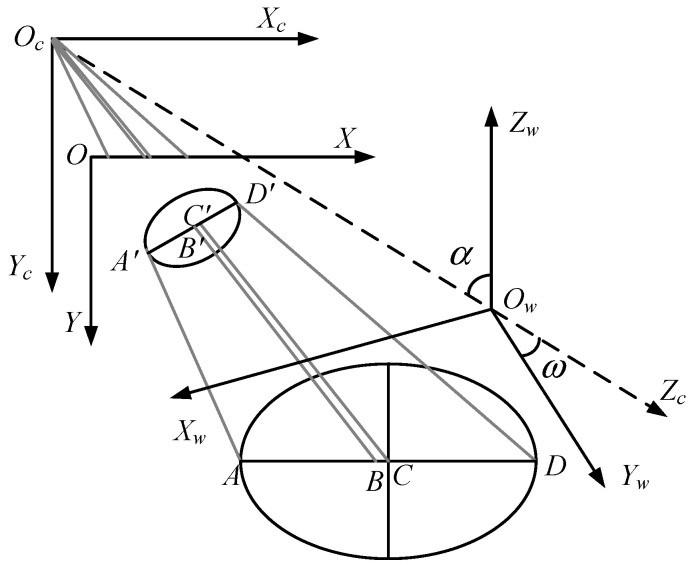
Schematic Diagram of the Perspective Projection of a Spatial Circle.

When the plane of a spatial circle is not parallel to the image plane of the camera, its projection onto the image plane appears as an ellipse. In practical applications, the geometric center of this ellipse is often mistakenly considered as the projected point of the spatial circle’s center. However, this assumption can introduce significant localization errors. As illustrated in the perspective projection diagram of the spatial circle in Figure 13, point $O_{c}$ denotes the optical center of the camera, and the camera coordinate system $O_{c}-X_{c}Y_{c}Z_{c}$ is established, with $O_{c}Z_{c}$ representing the camera’s optical axis. In the world coordinate system, a circle with radius *r* is defined, and its center is located at point C. After perspective projection through the camera, the circle appears as an ellipse on the image plane, with the ellipse center located at point $B^{\prime }$, while the true projection of the circle center appears at point $C^{\prime }$.

As shown in Figure 13, the projected point $C^{\prime }$ of the circle center does not coincide with the geometric center $B^{\prime }$ of the ellipse. If the geometric center of the ellipse is directly treated as the projected point of the circle center, it will inevitably introduce errors into the subsequent measurement and localization results [22].

### 3.2. Computation of the Normal Vector of the Spatial Circular Plane

**Figure 14:**
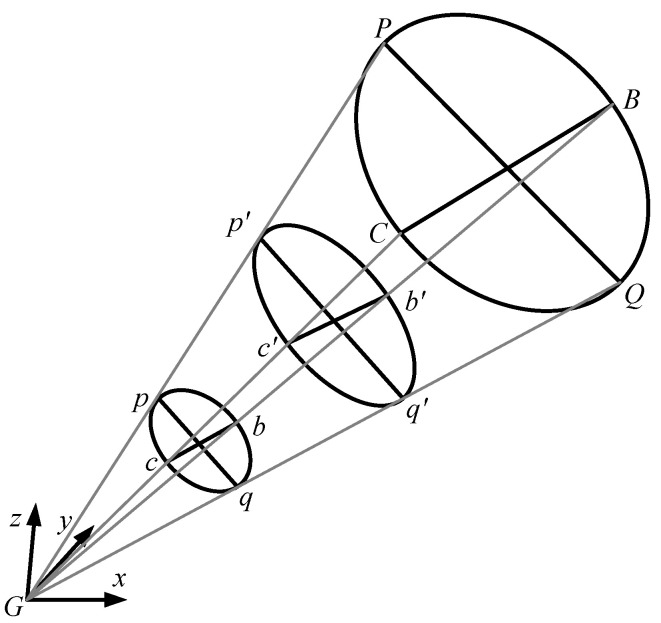
Schematic Diagram of the Perspective Projection of a Spatial Circle.

To obtain the actual projected point of the spatial circle center, the normal vector of the spatial circular plane must first be calculated. Taking the white circle of the fiducial marker as an example, the elliptical contour features of the marker are extracted from the left and right images through image processing. The ellipse contours are then discretized to obtain the pixel coordinates of the contour points. Let point *G* represent the optical center of the camera. By traversing the ellipse contour points, points *p* and *q* are identified such that the angle ∠pGq reaches its maximum. A plane *bGc* is then constructed, which is perpendicular to the plane *pGq* and bisects the angle ∠pGq. The intersections of plane *bGc* with the ellipse contour yield points *b* and *c*, as illustrated in Figure 14. According to reference [23], the formula for calculating the normal vector of the spatial circular plane is given in Equation (14).

(14) $$\left\{\begin{array}{l} n_{1}=(n_{x},n_{y},n_{z})=\frac{\left(0,f\sqrt{u_{p^{\prime }}^{2}-v_{b^{\prime }}^{2}},-v_{b^{\prime }}\sqrt{u_{p^{\prime }}^{2}+f^{2}}\right)}{\sqrt{(u_{p^{\prime }}^{2}-v_{b^{\prime }}^{2})f^{2}+(u_{p^{\prime }}^{2}+f^{2})v_{b^{\prime }}^{2}}}\\ n_{2}=(n_{x},-n_{y},n_{z}) \end{array}\right.$$

In the equation, $n_{1}$ and $n_{2}$ represent the two possible solutions for the normal vector of the spatial circular plane. Let *f* denote the focal length of the lens, and $u_{p^{\prime }}$ and $v_{b^{\prime }}$ can be calculated using Equation (15).

(15) $$\left\{\begin{array}{l} u_{p^{\prime }}=f\tan(∠(pGq/2))\\ v_{b^{\prime }}=f\tan(∠(bGc/2)) \end{array}\right.$$

Using binocular vision, two possible solutions, $n_{l1}$ and $n_{l2}$, for the normal vector can be obtained from the left camera image, while two possible solutions, $n_{r1}$ and $n_{r2}$, can be derived from the right camera image. Among each pair, one vector corresponds to the correct normal direction. However, due to measurement errors, the two correct normal vectors are not exactly identical. The angle between the normal vectors obtained from the left and right images is calculated as shown in Equation (16).

(16) $$\left\{\begin{array}{l} \theta_{1}=∠(n_{l1},n_{r1})=\arccos\frac{n_{l1}\cdot n_{r1}}{\left\|n_{l1}\right\|\cdot \left\|n_{r1}\right\|}\\ \theta_{2}=∠(n_{l1},n_{r2})=\arccos\frac{n_{l1}\cdot n_{r2}}{\left\|n_{l1}\right\|\cdot \left\|n_{r2}\right\|}\\ \theta_{3}=∠(n_{l2},n_{r1})=\arccos\frac{n_{l2}\cdot n_{r1}}{\left\|n_{l2}\right\|\cdot \left\|n_{r1}\right\|}\\ \theta_{2}=∠(n_{l2},n_{r2})=\arccos\frac{n_{l2}\cdot n_{r2}}{\left\|n_{l2}\right\|\cdot \left\|n_{r2}\right\|} \end{array}\right.$$

The minimum value $\theta_{\min}$ is selected from among $\theta_{1}$, $\theta_{2}$, $\theta_{3}$ and $\theta_{4}$, The normal vectors $n_{l}$ and $n_{r}$ corresponding to $\theta_{\min}$, are determined as the correct normal vectors for the left and right images, respectively. In this way, the dual-solution problem of the spatial circular plane’s normal vector is effectively resolved.

### 3.3. Computation of the Spatial Circle Center Coordinates

In 3D space, parallel planes intersect at infinity, and their intersection line is projected onto the image as the vanishing line of the plane. Given the camera’s intrinsic parameter matrix *M*, obtained through camera calibration, the vanishing line *L* of the spatial circular plane in the camera coordinate system can be computed using the formula shown in Equation (17).

(17) $$L=M^{-T}\cdot n$$

According to the fitted equation of the fiducial ellipse expressed in Equation (8), the parameter matrix *w* of the ellipse is defined as follows:

(18) $$w=\left(\begin{array}{ccc} A & B/2 & D/2\\ B/2 & C & E/2\\ D/2 & E/2 & F \end{array}\right)$$

The actual projected point *o* of the spatial circle center on the image plane is thus determined, as shown in [23]:

(19) $$o=w^{-1}\cdot L$$

The actual projected points $o_{l}$ and $o_{r}$ of the spatial circle center on the left and right camera images, respectively, are computed. Based on the binocular vision imaging model, the spatial coordinates of the circle center in the camera coordinate system are then calculated, thereby achieving precise localization of the spatial circle center.

### 3.4. Experimental Analysis of Precise Localization of the Spatial Circle Center

**Figure 15:**
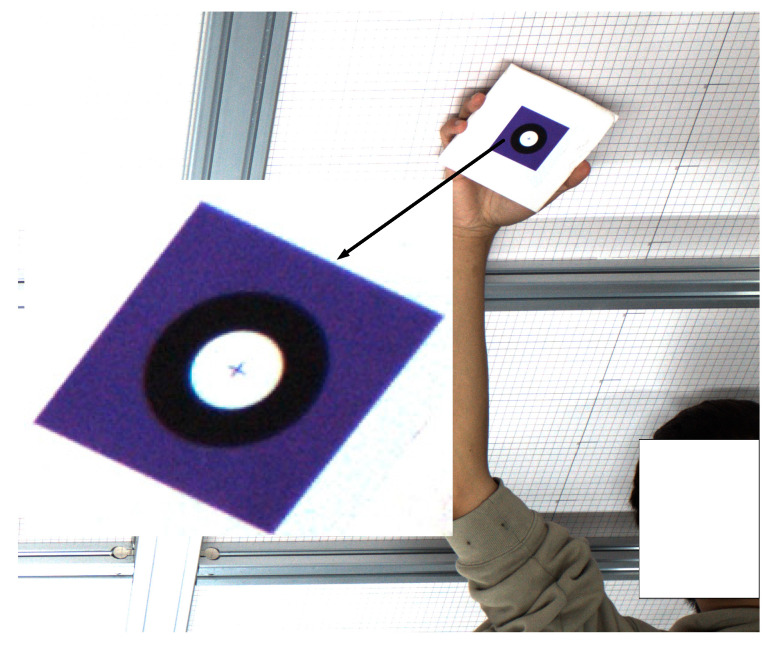
Fiducial Marker Images Captured within the Field of View of the Stereo Camera System.

To verify the effectiveness of the proposed algorithm, experimental validation and analysis were conducted. Under real-world industrial conditions, 30 fiducial marker images captured at various positions within the binocular camera’s field of view were selected for testing. As shown in Figure 15, the images of the fiducial markers within the binocular field of view are presented, where the cross mark indicates the ideal projection point of the center of the white circle in the marker.

The projection coordinates of the circle center were calculated using the method proposed in this paper. Table 2 lists a subset of the ideal projection points, the ellipse-fitted centers, and the computed projection coordinates of the circle center. Table 3 presents the error analysis data, showing the distances from both the ellipse-fitted centers and the computed projection points to the corresponding ideal projection points.

As shown by the data in Table 2 and Table 3, the distance error between the ellipse-fitted center and the ideal projection point is relatively large, with an average error of 2.5048 pixels. In contrast, the average error of the center coordinates computed using the proposed algorithm is reduced to 0.1230 pixels. This demonstrates that the circle center coordinates obtained by the proposed method are significantly closer to the ideal projection point, indicating the effectiveness of the approach.

**Figure 16:**
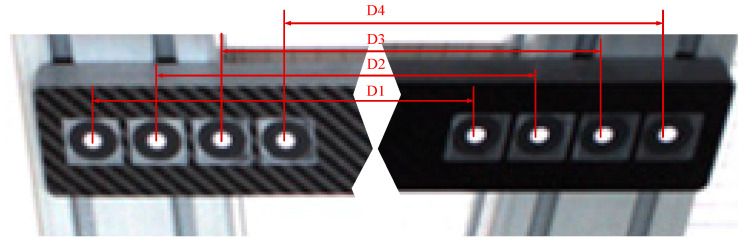
Schematic Diagram of the Calibration Rod.

**Figure 17:**
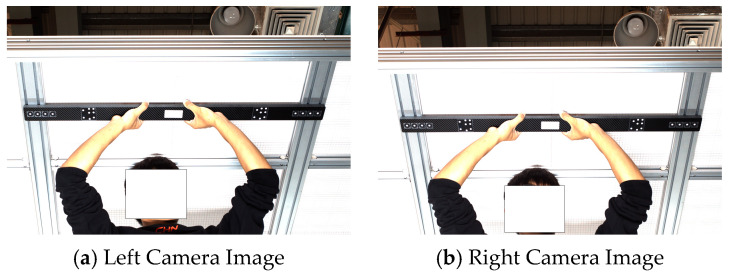
Reference Rod Image.

To further verify the effectiveness of the proposed algorithm, the binocular camera system was calibrated using the MATLAB toolbox. Through calibration, the intrinsic parameters of the binocular system, as well as the rotation and translation between the left and right cameras, were obtained. Based on the binocular stereo vision model [24], the 3D coordinates of the points were then computed. By calculating the Euclidean distances between the projected center points and comparing them with the theoretical lengths of a reference rod, the effectiveness of the proposed method was evaluated. A schematic diagram of the reference rod is shown in Figure 16, where the theoretical lengths of segments D1, D2, D3, and D4 are 882.546 mm, 882.753 mm, 882.473 mm, and 882.902 mm, respectively. The captured images of the reference rod are presented in Figure 17 with Figure 17a showing the left camera image and Figure 17b showing the right camera image.

A total of 10 reference rod images were captured within the field of view of the binocular camera system. The proposed algorithm was used to calculate the center pixel coordinates from both the left and right camera images. Table 4 lists a subset of the computed center pixel points and ellipse-fitted centers in the left camera images. DL represents the pixel coordinates of the left circle center corresponding to a given length, and DR represents the pixel coordinates of the right circle center. As shown in the table, there is a noticeable difference between the ellipse-fitted centers and the computed center pixel points.

**Figure 18:**
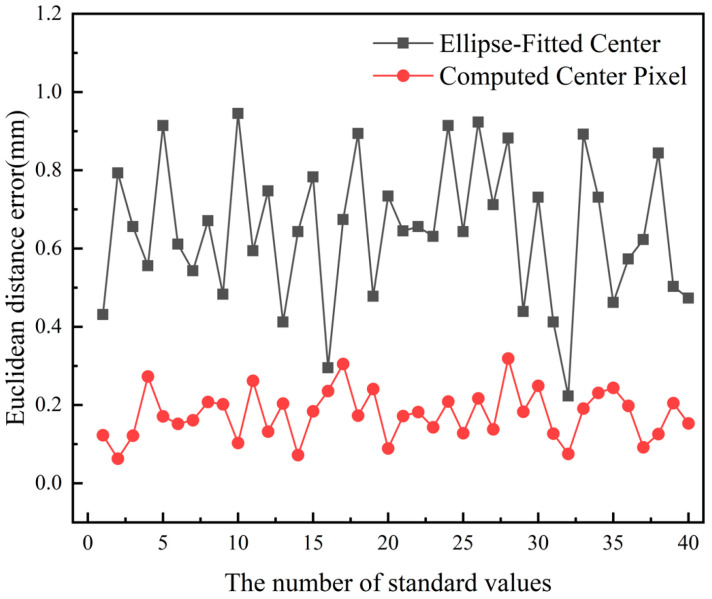
Comparison of Euclidean Distance Errors.

The 3D coordinates of the points were calculated using both the ellipse-fitted centers and the center pixel points obtained through the proposed method. The Euclidean distances between the resulting 3D points were then computed and compared with the standard values of the reference rod. Due to the large volume of data and space limitations, only a subset of the Euclidean distance error analysis results is presented in Table 5, while Figure 18 illustrates a comparison of Euclidean distance errors for all data samples.

The average Euclidean distance error of the center pixel points computed by the proposed algorithm was 0.176 mm, which is significantly lower than the average error of 0.644 mm obtained from the ellipse-fitted centers. Furthermore, both the maximum and minimum Euclidean distance errors of the center pixel points calculated by the proposed method were smaller than those of the ellipse-fitted centers. These results validate the effectiveness and accuracy of the center pixel computation method presented in this paper.

## 4. Experimental Validation and Analysis

**Figure 19:**
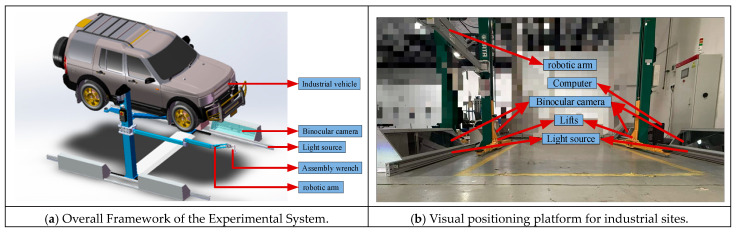
Visual positioning system.

In automotive chassis bolt localization tasks, a typical approach uses a single pair of stereo cameras to localize bolts on the chassis, which limits the system’s field of view and application range. This paper proposes a multi-camera stereo vision system for automotive chassis bolt localization by deploying four pairs of stereo cameras symmetrically on both sides of the automotive assembly station. The goal is to increase the system’s field of view and adaptability. The study focuses on automotive chassis bolts, where a robotic arm’s traction mechanism guides the assembly wrench near the bolt to be assembled. The stereo vision system is then used to spatially localize the fiducial marker on the bottom of the wrench. Based on the known spatial geometric relationship between the marker and the bolt end, the position parameters of the bolt end in the vehicle coordinate system are derived. These parameters are then matched with the tightening process parameters from the database, and the matched tightening parameters are sent to the assembly wrench for assembly. The overall framework of the experimental system is designed based on the above requirements, as shown in Figure 19a.

Based on the overall system structure plan, a corresponding visual localization experimental platform has been built, and its overall layout is shown in Figure 19b. The experimental platform mainly includes: a robotic arm, stereo cameras, lighting system, lifter, and computer. For the visual perception part, a Hikvision industrial camera (model: MV-CS200-10UC) is used to form the stereo camera system, with a matching lens model of MVL-KF1224M-25MP and a lens focal length of 12 mm. The lighting system employs a white diffuse strip light source to address the issue of insufficient lighting on automotive chassis in industrial environments. During the experiment, a double-column electric hydraulic lift is used to support and adjust the height of the automotive chassis. The computing and control unit is a computer equipped with an Intel Core i9-9750H processor and a 64-bit Windows 10 operating system, which is used for image processing, algorithm execution, and system control.

**Figure 20:**
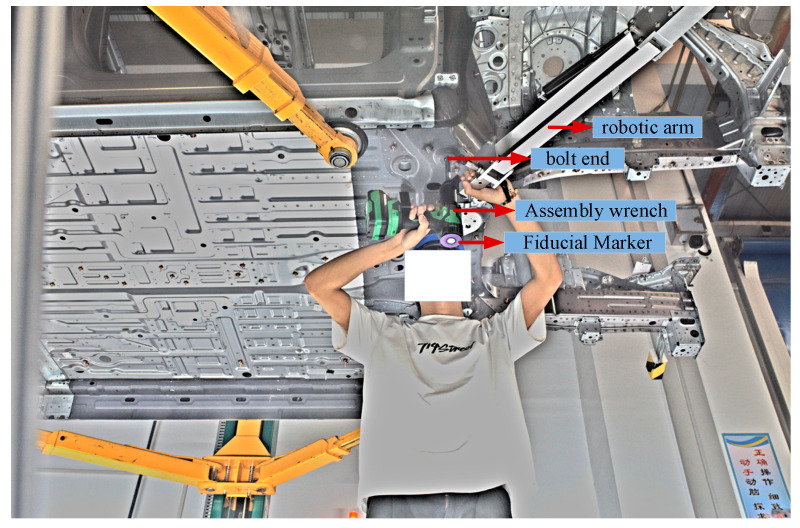
Bolt Localization Process.

In actual industrial conditions, the vehicle is fixed on the lift platform, and the chassis is raised to the effective working plane of the cameras through the lifting motion. Subsequently, the robotic arm pulls the assembly wrench to the target bolt position, and the stereo vision system is used to localize the automotive chassis bolt, obtaining the position parameters of the bolt end in the vehicle coordinate system. These parameters are then matched with the tightening process parameters from the database, and the matched tightening parameters are transmitted to the assembly wrench to complete the assembly. As shown in Figure 20, this is an image captured by the camera of the operator performing the automotive chassis bolt localization. It should be noted that when the wrench is perpendicular to the automotive chassis in the vehicle coordinate system, the center of the fiducial marker at the end of the assembly wrench has the same (X, Y) coordinates as the bolt end on the chassis. Using the algorithm presented in this paper, the actual projected center of the fiducial marker is calculated in the camera coordinate system. The coordinates of the marker center in the camera coordinate system are then transformed to the vehicle coordinate system using affine transformation [25]. This provides the (X, Y) coordinates of the marker center in the vehicle coordinate system, which corresponds to the (X, Y) coordinates of the bolt end in the vehicle coordinate system obtained through localization. Therefore, the (X, Y) coordinates of the bolt end obtained through localization are compared with the theoretical (X, Y) coordinates of the bolt hole to evaluate the system’s localization accuracy and verify the feasibility of the proposed visual bolt localization scheme.

### 4.1. Robustness Evaluation of Ellipse Fitting Under Disturbances

Strip light sources were positioned on both sides of the vehicle. When the vehicle was lifted to the working plane by the hoist, the illumination of the chassis was observed to be non-uniform due to the large structural size of the automotive chassis, exhibiting a gradual darkening trend from the center toward the edges. When the bolts to be localized were located in poorly illuminated regions, the corresponding fiducial marker images exhibited higher levels of noise. Therefore, the ellipse fitting algorithm proposed in this study must be capable of handling the increased noise present under low-light conditions. The following section evaluates the performance of the proposed algorithm under salt-and-pepper noise.

**Figure 21:**
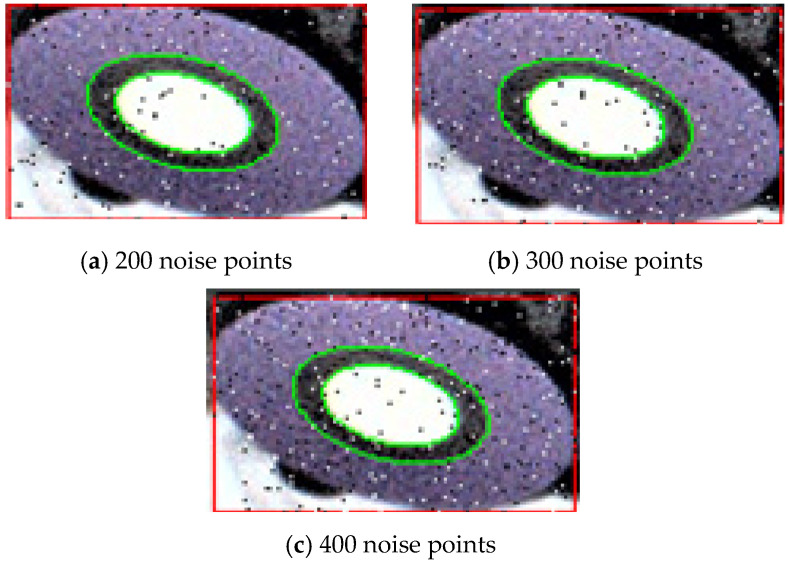
Experimental results under salt-and-pepper noise interference.

Salt-and-pepper noise is a type of random noise in which certain pixels in an image are randomly selected and their values replaced with either black or white. This type of noise is typically caused by communication errors during data transmission, where the values of some pixels may be lost, thereby degrading image quality [26]. In the experiment, 50 fiducial marker images were selected from various positions under the automotive chassis and at different angles relative to the camera. Within the region of interest (ROI) of the marker (measuring 120 pixels × 75 pixels), 200, 300, and 400 salt-and-pepper noise points were added, respectively, to evaluate the robustness of the proposed ellipse fitting algorithm. The experimental results under salt-and-pepper noise interference are shown in Figure 21.

Under salt-and-pepper noise interference, an error analysis was conducted on the proposed ellipse fitting algorithm. The standard deviation, mean error, maximum error, and minimum error of the estimated radii for the white circle and black ring were calculated. The error analysis results under salt-and-pepper noise are summarized in Table 6 Experimental results demonstrate that the proposed algorithm remains capable of accurately fitting the fiducial marker ellipses even in the presence of noise, maintaining high precision. These findings confirm that the visual localization method proposed in this study exhibits strong robustness and anti-interference capability under complex lighting conditions encountered in real industrial environments, thereby meeting the practical requirements for stability and accuracy in automated bolt assembly applications.

### 4.2. Analysis of Positioning Accuracy

Fifty bolts located at different positions and depths on the vehicle chassis were selected for positioning. The *X* and *Y* coordinates of the bolt endpoints obtained through the proposed localization method were compared with the theoretical *X* and *Y* coordinates of the corresponding bolt hole centers. The relative positioning errors were analyzed to determine whether the method satisfies the accuracy requirements of practical industrial assembly environments.

**Figure 22:**
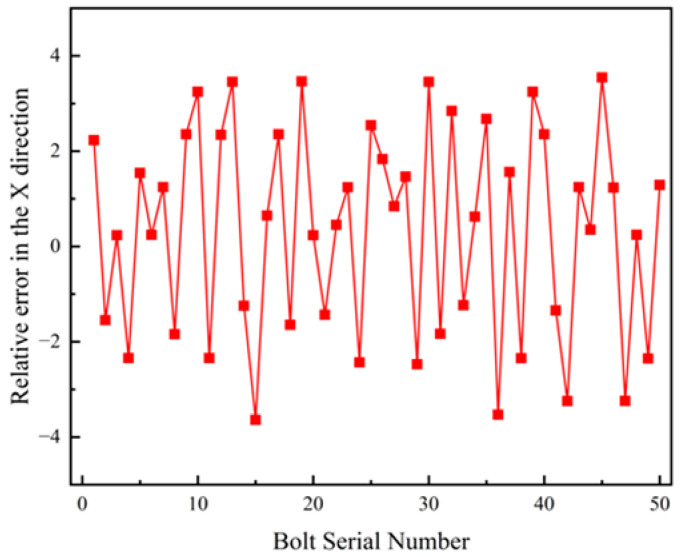
Schematic Diagram of Relative Error in the X Direction.

Figure 22 presents the line chart of relative errors along the *X* axis. The relative positioning error ranges from −3.642 mm to 3.545 mm, with a mean relative error of 1.884 mm and a variance of 1.236 mm^2^.

**Figure 23:**
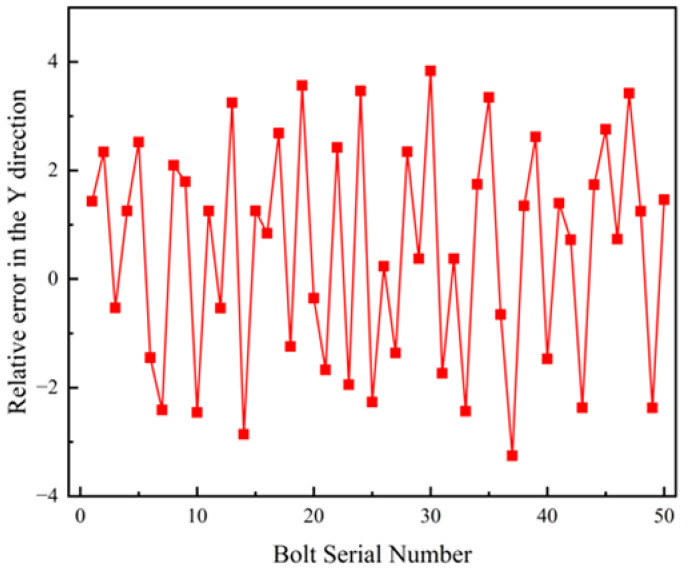
Schematic Diagram of Relative Error in the Y Direction.

Figure 23 presents the line chart of relative errors along the Y axis. The relative positioning error ranges from −3.225 mm to 3.834 mm, with a mean relative error of 1.844 mm and a variance of 1.001 mm^2^.

As observed from the figure, the positioning errors at the bolt endpoints exhibit a random distribution. This variability can be attributed to the non-uniform illumination conditions under the vehicle chassis and the operational randomness introduced by manual handling during each bolt positioning process. Nevertheless, all positioning errors remain within 4 mm, which meets the accuracy requirements of industrial assembly processes and demonstrates the method’s applicability in real-world production environments.

## 5. Conclusions

(1) To address the challenge of accurately locating bolts during automotive chassis assembly caused by non-uniform illumination, limited camera installation space, and structural occlusion, a fiducial-marker-based localization method for bolt assembly was proposed. An image processing pipeline was designed to extract the fiducial features on the bottom surface of the assembly wrench, comprising HSV color space segmentation, binarization, edge detection, contour connection, and ellipse fitting. In the color segmentation stage, an improved Retinex-based image enhancement algorithm was integrated to significantly improve the robustness and accuracy of region of interest (ROI) extraction under challenging lighting conditions. For ellipse fitting within the ROI, the Leitz criterion was introduced to optimize the fitting process. Experimental results demonstrated that the optimized ellipse fitting algorithm achieved higher accuracy and substantially improved fitting quality.
(2) Due to the perspective projection characteristics of spatial circles, a circular marker in 3D space is projected as an ellipse on the imaging plane of the camera, and the center of this ellipse does not correspond to the actual projection of the spatial circle’s center. A method for computing the true projection point of the spatial circle center on the image plane was investigated. Experimental results showed that the average error between the computed circle center and the ideal projection point was reduced from 2.5048 pixels to 0.1230 pixels using the proposed algorithm.
(3) Experimental results demonstrated that the proposed localization method for automotive chassis bolt assembly exhibited high stability and effectively addressed the issue of non-uniform illumination under the chassis. The algorithm showed strong anti-interference capability, and the localization results were verified by comparing them with the theoretical coordinates of the bolt holes. The achieved positioning accuracy was within millimeter-level precision, enabling high-accuracy localization of chassis bolts. This satisfies the precision requirements of industrial assembly processes and demonstrates the method’s applicability in real-world manufacturing environments.
(4) The localization method proposed in this paper has been validated in industrial environments and demonstrates high precision and robustness, meeting the localization requirements in the bolt assembly process. Additionally, the method has good scalability; it is not only applicable to automotive chassis bolt localization but can also be extended to other complex industrial assembly scenarios, such as part localization in electronic product assembly and component localization in aerospace assembly. Compared to learning-based algorithms, the method in this paper does not rely on large amounts of training data or complex models. Instead, it achieves precise localization through optimization strategies based on geometry and image processing, with relatively low computational cost. Furthermore, with the improvement of computational capabilities, this method is expected to integrate with deep learning technologies in the future, further enhancing its adaptability and intelligence, and expanding its application to localization tasks in more dynamic and complex environments.

## Figures and Tables

**Table 1:** Error Analysis of the Two Estimated Radii.

Radius/mm	Algorithm	Standard Deviation/μm	Mean Error/μm	Maximum Error/μm	Minimum Error/μm
10	Direct Least Squares Method	33.52	31.07	63	8
	3σ Method	23.83	22.23	48	6
15	Direct Least Squares Method	38.26	33.43	62	7
	3σ Method	28.50	25.00	52	6

**Table 2:** Selected Ideal Points, Ellipse-Fitted Centers, and Computed Projection Coordinates of Circle Centers (Unit: Pixels).

Index	Ideal Projection Point		Ellipse-Fitted Center		Computed Projected Center	
	*u*	*v*	*u*	*v*	*u*	*v*
1	421.51	1157.21	423.015	1158.853	421.423	1157.152
2	2485.241	1165.353	2483.858	1163.923	2485.205	1165.31
3	4825.682	1125.138	4827.035	1126.582	4825.542	1125.056
4	697.015	1969.842	695.834	1967.732	697.103	1969.973
5	2753.932	1977.632	2755.774	1979.326	2753.882	1977.612
6	4752.72	1971.562	4754.121	1973.982	4752.693	1971.483
7	841.215	2613.392	843.832	2614.932	841.204	2613.378
8	2737.842	2549.472	2735.026	2547.623	2737.762	2549.368
9	4533.632	2577.382	4535.012	2579.753	4533.512	2577.273
10	2777.173	3001.934	2775.923	2999.632	2777.036	3001.829

**Table 3:** Error Analysis of Distance Measurements (Unit: Pixels).

	Maximum Error	Minimum Error	Mean Error	Standard Deviation
Computed Projected Center	3.3688	1.9788	2.5048	0.1709
Computed Projected Center	0.1726	0.0483	0.1230	0.0022

**Table 4:** Ellipse-Fitted Centers and Computed Central Pixel Coordinates (Unit: Pixels).

Index	Length		Ellipse-Fitted Center		Computed Center Pixel	
1	D1	D_L_	1653.182	1251.272	1651.387	1249.374
		D_R_	2814.172	1244.3292	2812.434	1242.230
	D2	D_L_	1743.021	1250.892	1740.239	1249.132
		D_R_	4902.893	1244.062	4901.298	1241.834
	D3	D_L_	1831.082	1250.402	1829.378	1248.832
		D_R_	4992.182	1243.521	4990.321	1241.302
	D4	D_L_	1921.013	1249.739	1918.748	1247.924
		D_R_	5081.231	1242.832	5079.239	1240.893

**Table 5:** Partial Euclidean Distance Error Analysis Data (Unit: mm).

Standard Value	Ellipse-Fitted Center		Computed Center Pixel	
	Euclidean Distance	Error	Euclidean Distance	Error
882.546	882.115	0.431	882.423	0.123
882.546	882.053	0.493	882.383	0.163
882.753	882.297	0.456	882.631	0.122
882.753	882.197	0.556	882.583	0.170
882.473	881.959	0.514	882.302	0.171
882.473	881.862	0.611	882.323	0.150
882.902	882.359	0.543	882.742	0.160
882.902	882.231	0.671	882.694	0.208

**Table 6:** Error analysis of salt and pepper noise.

Radius/mm	Noise s10.	Standard Deviation/μm	Mean Error/μm	Maximum Error/μm	Minimum Error/μm
10	200	25.43	24.08	53	12
	300	26.72	25.15	58	14
	400	28.53	27.25	69	14
15	200	30.14	28.84	56	13
	300	31.57	30.45	66	13
	400	33.46	32.67	73	15

## Data Availability

All data and models generated or used during the study appear in the submitted article. Because some data of this paper will be used in the next research plan of the research group, the data sets generated and/or analyzed in this study are not public, but reasonable requirements can be obtained from the corresponding authors.
